# Corrigendum to: Recommendations to enhance rigor and reproducibility in biomedical research

**DOI:** 10.1093/gigascience/giaa103

**Published:** 2020-09-17

**Authors:** Jaqueline J Brito, Jun Li, Jason H Moore, Casey S Greene, Nicole A Nogoy, Lana X Garmire, Serghei Mangul

**Affiliations:** Department of Clinical Pharmacy, School of Pharmacy, University of Southern California, 1985 Zonal Avenue, Los Angeles, CA 90089, USA; Department of Computational Medicine & Bioinformatics, Medical School, University of Michigan, 1301 Catherine Street, Ann Arbor, MI 48109, USA; Department of Biostatistics, Epidemiology, and Informatics, Institute for Biomedical Informatics, University of Pennsylvania, 3700 Hamilton Walk, Philadelphia, PA 19104, USA; Department of Systems Pharmacology and Translational Therapeutics, Perelman School of Medicine, University of Pennsylvania, 3400 Civic Center Boulevard, Philadelphia, PA 19104, USA; Childhood Cancer Data Lab, Alex's Lemonade Stand, 1429 Walnut St, Floor 10, Philadelphia, PA 19102, USA; GigaScience, 26/F, Kings Wing Plaza 2, 1 On Kwan Street, Shek Mun, N.T., Hong Kong; Department of Computational Medicine & Bioinformatics, Medical School, University of Michigan, 1301 Catherine Street, Ann Arbor, MI 48109, USA; Department of Clinical Pharmacy, School of Pharmacy, University of Southern California, 1985 Zonal Avenue, Los Angeles, CA 90089, USA; Quantitative and Computational Biology, University of Southern California, Los Angeles, CA 90089, USA

In the original publication of this article, the author Serghei Mangul was erroneously listed with a second affiliation: Quantitative and Computational Biology, University of Southern California, Los Angeles, CA, 90 090, USA, which has been removed from the author byline. The authors regret this error.

