# Supplementary material for: Recommendations to enhance rigor and reproducibility in biomedical research
Source: Gigascience. 2020 Jun 1;9(6):giaa056. doi: 10.1093/gigascience/giaa056 (PMC7263079; doi:10.1093/gigascience/giaa056)
Supplement: giaa056_GIGA-D-20-00054_Original_Submission [file giaa056_giga-d-20-00054_original_submission.pdf]

GigaScience

Recommendations to enhance rigor and reproducibility in biomedical research

--Manuscript Draft--

**Manuscript Number:**	GIGA-D-20-00054
**Full Title:**	Recommendations to enhance rigor and reproducibility in biomedical research
**Article Type:**	Commentary
**Funding Information:**	National Institutes of Health (R01HG010067) — PhD Casey S. Greene<br>National Institutes of Health (R01CA237170) — PhD Casey S. Greene<br>Gordon and Betty Moore Foundation (US) (GBMF4552) — PhD Casey S. Greene<br>Silicon Valley Community Foundation (2018-182718) — PhD Casey S. Greene<br>National Institute of Environmental Health Sciences (K01ES025434) — PhD Lana X. Garmire<br>U.S. National Library of Medicine (R01 LM012373) — PhD Lana X. Garmire<br>U.S. National Library of Medicine (R01 LM012907) — PhD Lana X. Garmire<br>Eunice Kennedy Shriver National Institute of Child Health and Human Development (R01 HD084633) — PhD Lana X. Garmire
**Abstract:**	Computational methods have reshaped the landscape of modern biology. While the biomedical community is increasingly dependent on computational tools, the mechanisms ensuring open data, open software, and reproducibility are variably enforced by academic institutions, funders and publishers. Publications may describe the software for which source code is unavailable, documentation is incomplete or unmaintained, and analytical source code is missing. Publications that lack this information compromise the role of peer review in evaluating technical strength and scientific contribution. Such flaws also limit any subsequent work that intends to use the described software. We herein provide recommendations to improve reproducibility, transparency, and rigor in computational biology—precisely the values which should be emphasized in foundational life and medical science curricula. Our recommendations for improving software availability, usability, and archival stability aim to foster a sustainable data science ecosystem in biomedicine and life science research.
**Corresponding Author:**	Jaqueline Joice Brito<br>University of Southern California<br>Los Angeles, UNITED STATES
**Corresponding Author Secondary Information:**	
**Corresponding Author's Institution:**	University of Southern California
**Corresponding Author's Secondary Institution:**	
**First Author:**	Jaqueline J. Brito
**First Author Secondary Information:**	
**Order of Authors:**	Jaqueline J. Brito<br>Jun Li<br>Jason H. Moore<br>Casey S. Greene<br>Nicole A. Nogoy

Powered by Editorial Manager® and ProduXion Manager® from Aries Systems Corporation

	Lana X. Garmire
	Serghei Mangul
**Order of Authors Secondary Information:**	
**Additional Information:**	
**Question**	**Response**
Are you submitting this manuscript to a special series or article collection?	No
**Experimental design and statistics**<br><br>Full details of the experimental design and statistical methods used should be given in the Methods section, as detailed in our Minimum Standards Reporting Checklist. Information essential to interpreting the data presented should be made available in the figure legends.<br><br>Have you included all the information requested in your manuscript?	No
If not, please give reasons for any omissions below.<br><br>as follow-up to "**Experimental design and statistics**<br><br>Full details of the experimental design and statistical methods used should be given in the Methods section, as detailed in our Minimum Standards Reporting Checklist. Information essential to interpreting the data presented should be made available in the figure legends.<br><br>Have you included all the information requested in your manuscript?<br><br>"	Not applicable.
**Resources**<br><br>A description of all resources used, including antibodies, cell lines, animals and software tools, with enough	No

Powered by Editorial Manager® and ProduXion Manager® from Aries Systems Corporation

information to allow them to be uniquely identified, should be included in the Methods section. Authors are strongly encouraged to cite Research Resource Identifiers (RRIDs) for antibodies, model organisms and tools, where possible.<br><br>Have you included the information requested as detailed in our Minimum Standards Reporting Checklist?	
If not, please give reasons for any omissions below.<br><br>as follow-up to "**Resources**<br><br>A description of all resources used, including antibodies, cell lines, animals and software tools, with enough information to allow them to be uniquely identified, should be included in the Methods section. Authors are strongly encouraged to cite Research Resource Identifiers (RRIDs) for antibodies, model organisms and tools, where possible.<br><br>Have you included the information requested as detailed in our Minimum Standards Reporting Checklist?<br><br>"	Not applicable.
**Availability of data and materials**<br><br>All datasets and code on which the conclusions of the paper rely must be either included in your submission or deposited in publicly available repositories (where available and ethically appropriate), referencing such data using a unique identifier in the references and in the "Availability of Data and Materials" section of your manuscript.<br><br>Have you have met the above requirement as detailed in our Minimum	No

Standards Reporting Checklist?	
If not, please give reasons for any omissions below.<br><br>as follow-up to "**Availability of data and materials**<br><br>All datasets and code on which the conclusions of the paper rely must be either included in your submission or deposited in publicly available repositories (where available and ethically appropriate), referencing such data using a unique identifier in the references and in the "Availability of Data and Materials" section of your manuscript.<br><br>Have you have met the above requirement as detailed in our Minimum Standards Reporting Checklist?<br><br>"	There is no dataset associated with this paper.

Manuscript Click here to download Manuscript Manuscript.pdf

{Commentary}

Recommendations to enhance rigor and reproducibility in biomedical research

Jaqueline J. Brito[1,*], Jun Li[2], Jason H. Moore[3], Casey S. Greene[4,5], Nicole A. Nogoy[6$], Lana X. Garmire[2$], Serghei Mangul[1,7,$*]

[1] Department of Clinical Pharmacy, School of Pharmacy, University of Southern California, 1985 Zonal Avenue Los Angeles, CA 90089, USA

[2] Department of Computational Medicine & Bioinformatics, Medical School, University of Michigan, 1301 Catherine St. Ann Arbor, MI 48109, USA

[3] Department of Biostatistics, Epidemiology, and Informatics, Institute for Biomedical Informatics, University of Pennsylvania, 3700 Hamilton Walk, Philadelphia, PA 19104, USA

[4] Department of Systems Pharmacology and Translational Therapeutics, Perelman School of Medicine, University of Pennsylvania, 3400 Civic Center Blvd, Philadelphia, PA 19104, USA

[5] Childhood Cancer Data Lab, Alex's Lemonade Stand, Philadelphia, PA 19102, USA

[6] GigaScience, 26/F, Kings Wing Plaza 2, 1 On Kwan Street, Shek Mun, N.T, Hong Kong

[7] Quantitative and Computational Biology, University of Southern California, Los Angeles, CA, 90089, USA

$ - These authors contributed equally to the paper

*Correspondence: britoj@usc.edu; serghei.mangul@gmail.com

Abstract

Computational methods have reshaped the landscape of modern biology. While the biomedical community is increasingly dependent on computational tools, the mechanisms ensuring open data, open software, and reproducibility are variably enforced by academic institutions, funders and publishers. Publications may describe the software for which source code is unavailable, documentation is incomplete or unmaintained, and analytical source code is missing. Publications that lack this information compromise the role of peer review in evaluating technical strength and scientific contribution. Such flaws also limit any subsequent work that intends to use the described software. We herein provide recommendations to improve reproducibility, transparency, and rigor in computational biology—precisely the values which should be emphasized in foundational life and medical science curricula. Our recommendations for improving software availability, usability, and archival stability aim to foster a sustainable data science ecosystem in biomedicine and life science research.

Keywords

Rigor, reproducible research, installability, archival stability, big data, open science

Main text

Biomedical informatics has the opportunity to be at the forefront of the community in developing practices that promote open data, open software, and reproducible research. The computational reproduction of previously published results is enabled when scientists publicly release all research resources, from raw data to installable packages and source code, in a discoverable and archivally stable manner. Publications lacking data or source code undermine scientific rigor, transparency, and reproducibility[1]. Platforms already exist that support public release of scientific materials, but the lack of strict enforcement by journals, academic institutions, and funding agencies has resulted in the loss of crucial research objects for many published studies.

An astonishing number of bioinformatics software tools are designed each year to accommodate increasingly bigger, more complex, and more specialized biomedical datasets[2]. Many of those software tools have limited installability and are hosted on Uniform Resource Locators (URLs) with undetermined archiving practices[3]. Closed-source software presents unique challenges to reproducibility as researchers may not have access to the source code. Lack of access to the source code of a software package does not support the auditing of methods and results, and ultimately harms the transparency of research. We identify and discuss a pressing need for scientists to improve software availability, usability, and archival stability in computational

biology. By following a set of best practices[4], scientists can promote rigor and reproducibility, ultimately cultivating a sustainable, thriving research community.

1. Increase computational training opportunities targeted at reproducibility. Biomedical researchers who use computational tools must acquire specific computational skills in order to successfully apply the techniques to a large amount of data. Undergraduate students who lack formal computational training can be taught the skills required to promote reproducibility via specialized courses. In addition to rigorous class training, advanced undergraduate and graduate students may benefit from short-term intensive workshops aimed at postdoctoral scholars, clinical fellows, and faculty. Several institutions, including the University of California, Los Angeles, have successfully hosted workshop-based programs for over five years and serve as valuable resources for pedagogy and curricula[5]. Workshops for training researchers to use computational tools usually include hands-on training for implementing analysis tools, such as cloud-based notebook technologies. Since 1998, Software Carpentry (https://software-carpentry.org/) has been holding volunteer-based training courses for researchers with the computational skills required to keep up with the demands of data- and computational-intensive research. Due to the reliance of today's analytical software packages on use of the command line, comprehensive computational training programs are ideal methods for training future life science and biomedical researchers towards reproducibility (**Figure 1a**).

2. Make all data and metadata open and discoverable. Open source code depends on the availability of open and shareable data. Access to the data used to produce important research results is key for auditing the rigor of published studies. Open access to datasets is imperative to building a thriving and sustainable scientific community where all researchers can access and analyze existing data. In practice, omics data of patients often cannot be shared publicly due to patient privacy and/or user agreement standards[6]. While not all data are freely publicly available, many studies provide controlled access, where researchers can access the raw data if they are approved after providing scientific rationale for access and signing user agreements. Also, in many cases where raw data are not accessible by the public, summary data are available. In general, the global data sharing climate has shifted towards a positive direction.

Sharing data can enable the reproducibility and robustness of science because of our ability to utilize data generated from individual studies to a larger scale. In addition, secondary analysis is economically sustainable and can be adopted as standard practice by scientists in countries with limited resources[7]. Ideally, data should also be discoverable via centralized repositories, such as Sequence Read Archive (SRA) and Gene Expression Omnibus (GEO), and annotated with descriptive metadata to enhance data reuse (**Figure 1b**). When data is shared on centralized repositories in interoperable formats, other researchers can examine and re-analyze the data, challenge existing interpretations, and test new theories. Data sharing corresponds to the true spirit of science, where each new discovery is built upon previous work and ultimately allows us to "stand on the shoulders of giants". Many important scientific discoveries have been solely based on shared data (e.g., economics, meteorology, and physics). In general, reusing the data

speaks about the quality and importance of generated data and contributed to the impact of the original work.

3. Build and use open-source software. Software provides a foundation for the potential reproducibility of published biomedical research, defined as the ability to replicate published findings by running the same computational tool on data generated by the study[4]. For this reason, closed-source and proprietary software restricts the reproducibility of biomedical research. First, researchers may not have access to the source code, which limits other researchers' ability to audit results and reviewers' ability to test the reproducibility prior to publication. Second, license restrictions may prohibit the creation of new functionalities that could be released on modified versions of existing tools. The cost of acquisition and maintenance of proprietary software licenses is not affordable for every laboratory. Reviewers may also not have access to proprietary software, and thus, may be unable to fully test the reproducibility of results. Therefore, the adoption of standard open-source licenses for data and software tools can enhance the rigor and impact of research by allowing any researcher to reproduce published studies.

Publicly releasing the source code does not guarantee the computational reproducibility of biomedical research. The open code must be well documented with user manuals and installable in a user-friendly manner. The code used in a published analysis should be hosted on an archivally stable platform such as GitHub (**Figure 1c**). Currently, over a quarter of computational software resources cannot be accessed through the URLs provided in the original publication, suggesting that the repositories are poorly maintained[3]. Additionally, many

bioinformatics tools are too difficult, or even impossible, for a new user to install[3]. Use of Open Source Initiative license models (https://opensource.org/licenses) allows users to easily use and adapt tools, increasing the sustainability of the biomedical research community. New platforms are also being proposed, such as CODE CHECK (https://sje30.github.io/codecheck/), where researchers can, input their code and data, and check that their analysis is reproducible. For verified analyses, CODE CHECK issues a time-stamped "*certificate of reproducible computation*" which can inform the peer reviewer of a paper.

4. Use platforms that enhance reproducibility. In addition to software and datasets, computational biology researchers commonly produce resources such as experiment protocols, workflows, and annotations. Storing and sharing these resources allows other researchers to cite them within a publication, which would increase the reproducibility of a paper and the visibility of previously developed methods. The inclusion of citable digital object identifiers (DOIs) also facilitates the discovery of reusable resources as they provide long-term access to published resources. Several innovative platforms designed to promote reproducibility have recently emerged (**Figure 1d**).

5. Make tools and workflows reproducible. To facilitate the reproducibility of tools it is good practice to utilize virtual machines (VMs) and containers. VMs are software pieces that are capable of encapsulating entire operating systems, libraries, codes, and data. For workflows, it is good practice to utilize workflow-specific platforms to enhance reproducibility, such as Galaxy or Bioconda, and workflow standards, such as CWL (Common Workflow Language) **(Table 1**).

There are now a myriad of platforms and tools available that make research reproducible and are already commonly used by life science and biomedical researchers (**Table 1**). Given the many different tools and platforms available that can promote reproducibility, a research lab should define their own standards on a suite of tools and platforms that support their research practices (**Figure 1e**).

Table 1: Examples of tools and platforms to share reproducible resources.

**Platform & Type**	**Use**
**Reproducible and open methods**	**Protocols.io** is an open-source protocol repository, where researchers can manage, share, tweak, optimize, and adopt existing methods even after a scientist has left a lab.
**RRIDs**	**Scicrunch.org** is a platform for curating research resources that enables the user to discover, access, view, and use research objects. Users can register any research object, such as tools, antibodies, animal models, etc. In turn, these objects are issued an RRID (Research Resource ID) which should be cited in the manuscript. The RRID allows other users to easily locate and access the resources.
**Annotations**	**Hypothes.is** is an open-source annotation tool that allows any researcher to annotate any resource on the web, including to

	annotate for personal use or as part of conversations available to private groups or the general public.
**Virtual Machines & Containers**	Containers such as **Docker** (www.docker.com), and **Singularity** (singularity.lbl.gov) are lightweight solutions compared to VMs as they do not encapsulate the operating system; rather, they rely on the host kernel to run required functions. Both VMs and containers are shared via image files and can be included as supplementary material at certain journals or stored in Zenodo (https://zenodo.org/), Figshare (https://figshare.com/), or other general-purpose archival repositories.
**Reproducible workflows**	**Galaxy** (https://galaxyproject.org/) is a computational platform which allows users to share workflows, histories, and wrapped tools in an easy-to-use and open source interface that even people without coding experience can use.<br>**Conda** (https://conda.io/) is a powerful open source package and management system that can quickly install, run and, update packages and their dependencies.<br>**Bioconda** (https://bioconda.github.io/) leverages Conda and is a community project and package manager dedicated to computational tools used by life science and biomedical researchers.

	**Common workflow language (CWL),** (https://www.commonwl.org) is an open standard used to describe workflows and tools to make them portable and interoperable across different environments (e.g., cloud, cluster or high-performance computing).
**Reproducible documents & Figures**	**Stencila** (https://stenci.la/) is an open source framework for executable documents and living figures (using R scripts). It supports commonly used environments and tools, such as Juptyer notebook, RMarkdown, Python, and SQL.<br>**Jupyter Notebook** (https://jupyter.org/) allows for the creation of sharing of live code, equations, visualizations, and narrative text. The application supports over 40 different programming languages and can be used to leverage big data.<br>**MyBinder** (https://mybinder.org/) is an application that collects and 'binds' interactive jupyter notebooks into a Binder repository and can also create a Docker image of that.

6. Make living and reproducible figures and papers. Open data and code is an important fundamental step toward transparency; however, over the last 5 years, it has been possible to break away from the static presentation of results and produce dynamic/living figures (**Figure 1f**). Dynamic figures allow a reader to alter the parameters as the code is actively running - an

iterative process where a figure can evolve as new data is added.One such example is Stenci.la, a platform that supports executable documents, living figures, and Jupyter Notebooks (**Table 1**).

7. Publish with journals that promote reproducibility. Journals have various publishing standards. A group of stakeholders, from academia and industry, defined a set of principles stating that research data should be Findable, Accessible, Interoperable, and Reusable (FAIR)[8]. Researchers may elect to publish in journals that encourage best practices (e.g., adopting the FAIR principles[8]) that aim to increase the impact of their work (**Figure 1g**). To ensure reproducibility, many journals now require that biomedical data generated by a published study be shared when the paper is released. For instance, *GigaScience* (gigasciencejournal.com) has been promoting reproducibility of analyses since 2012 (in addition to publishing open access) by mandating open data that follow the FAIR principles and mandates source code with an OSI approved license. During peer review, *GigaScience* also makes all supporting data and code available for Reviewers, and the editors ask the reviewers to test provided materials for reproducibility. Papers can aid this task by including VMs, containers, Jupyter notebooks, or packaged workflows (as opposed to static versions of these resources). Other journals, such as *Biostatistics*, have begun issuing badges for articles with validated data and code sharing**.** In 2018 *eLife* published their first example of a dynamic and code-based (using the Stencila platform, **Table 1**) reproducible article. This approach enables data and analysis to be fully reproducible by the reader and challenges the traditional static representation of results using PDF or HTML formats.

8. Support reusable resources. Successfully implementing and widely distributing software tools developed in academia involves unique challenges when compared to doing so in industry. In academia, software tools are developed by small groups comprised of graduate or postdoctoral scholars, who have fairly fast turn-over rates of 2-5 years. These groups are less likely to be professionally trained in software production standards when compared to software development groups in the industry, where holistic teams of specialists support the long-term maintenance of projects. In order to enhance the quality and reuse of open software, professionally trained software engineers should be hired to partner with the students and postdocs. To make this happen, funding agencies need better mechanisms of acknowledging and incentivizing funding earmarked for critical bioinformatics infrastructure (**Figure 1h**). In addition, funders should recognize the rigor of software development, rather than just considering 'novelty'-based conventional criteria of research. The availability of well-resourced grant mechanisms to convert minimum viable products produced by trainees into reliable software could enhance the impact of research-grade software on the community. With the growing number of biomedical datasets open for reuse in the public domain, It is inspiring to see the encouragement and acknowledgment of data reuse and secondary analysis with the research Parasite Awards[9]**.** The annual Parasite Awards recognizes the exceptional contributions for rigorous secondary analysis of data with recognition of the top-performing junior parasite and continued research parasite. More incentives, such as this, are required for software reuse.

Conclusions

We outlined key recommendations to improve the rigor of biomedical studies and foster reproducibility in computational biology. The infrastructure required to systematically adopt best practices for reproducibility of biomedical research is largely in place. The remaining challenge to the systemic promotion of reproducibility is that incentives are not currently aligned to support good practices. Instead, current efforts rely on individual researchers electing to follow best practices, often at their own time and expense. We believe it is time for a fundamental cultural shift in the scientific community: rigor and reproducibility should become primary concerns in the criteria and decision-making process of designing studies, funding research, and writing and publishing results. Successful systematic adoption of best practices will require the buy-in of multiple stakeholders in the scientific communities, from publishers, academic institutions, funding agencies, and stakeholders. This increases the lifetime and value of published research as resources naturally become reusable, testable, and discoverable. Community-wide adoption of best practices for reproducibility is critical to realizing the full potential of fast-paced, collaborative analyses of large datasets in the biomedical and life sciences. A dynamic version of this paper with an extended list of references was compiled in markdown and is available at[10].

Abbreviations

CWL, Common Workflow Language; DOI, Digital Object identifier; FAIR, Findable, Accessible, Interoperable, and Reusable; GEO, Gene Expression Omnibus; OSI, Open Source Initiative; RRID, Research Resource ID; SRA, Sequence Read Archive; URLs, Uniform Resource Locators; VM, Virtual Machine.

Competing interests

The authors declare that they have no competing interests.

Funding

CSG was supported by grants from the NIH (R01HG010067 and R01CA237170), the Gordon and Betty Moore Foundation (GBMF4552), the Chan Zuckerberg Initiative Donor Advised Fund of the Silicon Valley Community Foundation (2018-182718), and Alex's Lemonade Stand Foundation (CCDL). LXG is supported by grants K01ES025434 awarded by NIEHS through funds provided by the trans-NIH Big Data to Knowledge (BD2K) initiative (http://datascience.nih.gov/bd2k), R01 LM012373 and R01 LM012907 awarded by NLM, and R01 HD084633 awarded by NICHD. The funding bodies had no role in the design of the study and collection, analysis, and interpretation of data and in writing the manuscript.

Authors' contributions

Author	Contribution
JJB	Writing - Original Draft Preparation, review and editing.
JL	Writing - review and editing.
CSG	Writing - review and editing.
JM	Writing - review and editing.

NN	Conceptualization and structure of the manuscript; Writing - review and editing; Visualization - creation of Figures and Table.
LG	Conceptualizing the project; Writing - review and editing.
SM	Conceptualization and structure of the manuscript; Writing - review and editing.

Acknowledgments

We thank Dr. Lana Martin for discussions and helpful comments on the manuscript.

Authors' Information

NAN is an Editor at *GigaScience* and is an open science advocate with over 8 years experience in publishing reproducible research.

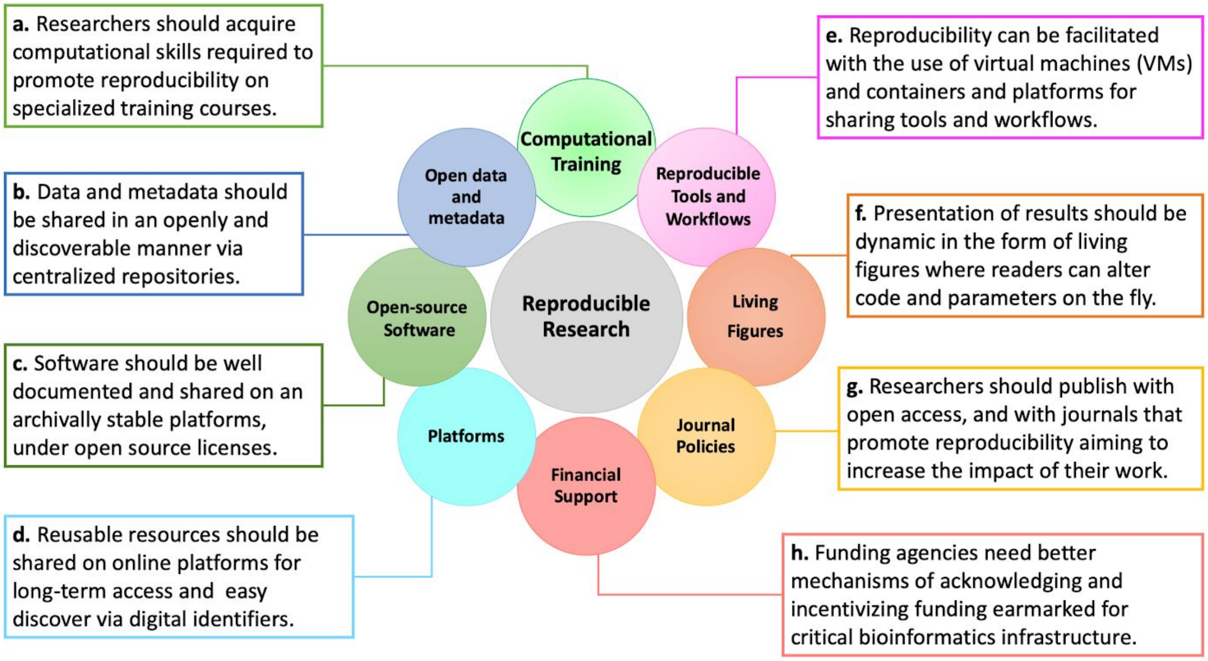

Figure 1. Recommendations to improve reproducibility and rigor of biomedical research.

References Cited

1. Stodden, V., Seiler, J. & Ma, Z. An empirical analysis of journal policy effectiveness for computational reproducibility. *Proc. Natl. Acad. Sci. U. S. A.* **115**, 2584–2589 (2018).
2. Wren, J. D. Bioinformatics programs are 31-fold over-represented among the highest impact scientific papers of the past two decades. *Bioinformatics* **32**, 2686–2691 (2016).
3. Mangul, S. *et al.* Challenges and recommendations to improve the installability and archival stability of omics computational tools. *PLoS Biol.* **17**, e3000333 (2019).
4. Piccolo, S. R. & Frampton, M. B. Tools and techniques for computational reproducibility.

GigaScience vol. 5 (2016).

5. Mangul, S., Martin, L. S., Hoffmann, A., Pellegrini, M. & Eskin, E. Addressing the Digital Divide in Contemporary Biology: Lessons from Teaching UNIX. *Trends Biotechnol.* **35**, 901–903 (2017).
6. Gewin, V. *et al.* Data sharing and the future of science. *Nat. Commun.* **9**, 1–2 (2018).
7. Mangul, S. *et al.* How bioinformatics and open data can boost basic science in countries and universities with limited resources. *Nat. Biotechnol.* **37**, 324–326 (2019).
8. Wilkinson, M. D. *et al.* The FAIR Guiding Principles for scientific data management and stewardship. *Sci Data* **3**, 160018 (2016).
9. Greene, C. S., Garmire, L. X., Gilbert, J. A., Ritchie, M. D. & Hunter, L. E. Celebrating parasites. *Nature genetics* vol. 49 483–484 (2017).
10. Brito, J. J. *et al.* Additional references for 'Recommendations to enhance rigor and reproducibility in biomedical research'. *GitHub* https://github.com/Mangul-Lab-USC/enhancing_reproducibility - Accessed Feb 21, 2020.

USC University of Southern California

USC SCHOOL OF PHARMACY
1450 Alcazar Street
Los Angeles, CA 90089

February 24, 2020

Dear Editors of GigaScience,

We are delighted to submit our manuscript titled "Recommendations to enhance rigor and reproducibility in biomedical research", which we hope will be a strong candidate for consideration as a Commentary paper at GigaScience.

Problem

Computational methods have reshaped the landscape of modern biology. While the biomedical community is increasingly dependent on computational tools, the mechanisms ensuring open data, open software, and reproducibility are variably enforced by academic institutions, funders and, publishers. Publications may describe the software for which source code is unavailable, documentation is incomplete or unmaintained, and analytical source code is missing. Publications that lack this information compromise the role of peer review in evaluating technical strength and scientific contribution. Such flaws also limit any subsequent work that intends to use the described software.

Solution

We provide recommendations to improve reproducibility, transparency, and rigor in computational biology -- precisely the values which should be emphasized in foundational life and medical science curricula. Our recommendations for improving software availability, usability, and archival stability aim to foster a sustainable data science ecosystem in biomedicine and life science research.

Sincerely,

Jaqueline J. Brito
Postdoctoral researcher
University of Southern California

Serghei Mangul
Assistant professor
University of Southern California
