# Supplementary material for: Recommendations to enhance rigor and reproducibility in biomedical research
Source: Gigascience. 2020 Jun 1;9(6):giaa056. doi: 10.1093/gigascience/giaa056 (PMC7263079; doi:10.1093/gigascience/giaa056)
Supplement: giaa056_Response_to_Reviewer_Comments_Original_Submission [file giaa056_response_to_reviewer_comments_original_submission.pdf]

Author's Response To Reviewer Comments

Close

Dear Dr. Scott Edmunds,

Thank you very much for considering our paper, entitled “Recommendations to enhance rigor and reproducibility in biomedical research,” for publication at GigaScience. We are grateful to the reviewer for their careful assessment of the manuscript and the detailed and constructive comments that they provided. Following the Board Member’s suggestions, we have implemented structural changes to improve clarity of the manuscripts. We also added generalisable and mature examples to illustrate concepts introduced in the manuscript. We believe that addressing these comments produced a much stronger manuscript that will hold more value for the scientific community.

Following the editor’s and reviewers’ comments, we have made changes listed below as a point-by-point response.

Sincerely,

Serghei Mangul, Ph.D.
University of Southern California

Response to Reviewers' Comments

Reviewer Comment #1: This paper aims to lay out a set of recommendations for enhancing the rigor and reproducibility of research across biomedicine. There is not much to specifically disagree with in the paper, and there are some points that I really like, such as the point that closed source/commercial software impairs reproducibility, the need for reproducible workflows, and the need for software engineers as part of research teams. At the same time, I don't feel that the paper really adds much to what is already out there - for example, in the many "ten simple rules" papers in PLOS Computational Biology. I also don't feel like the paper is organized very well; it seems to jump around quite a bit, and never gets into enough detail to really nail any of its points. I would suggest that the authors narrow their focus and build a clearer explication of their points in more detail.

Authors’ Response: We thank the reviewer for these valuable comments. We agree with the reviewer that we present discussions similar to the existing literature on reproducible computational research. However, we believe our paper goes further on the debate, adding other essential aspects that can help to improve reproducibility, such as the need for computational training, journal guidelines, and financial support. We have changed the text in the Introduction as follows in order to highlight how our paper differs from existing literature.

“Prior studies4 have addressed issues of computational reproducibility, including the need to automatize all data manipulation tasks and version control of code. We expand upon existing dialogue and emphasize reproducible research as computational training, journal policies, and financial support. We identify and discuss eight key recommendations across four different domains (Figure 1) to tackle the pressing need for scientists to improve software availability, usability, and archival stability in computational biology. By following a set of best practices5, scientists can promote rigor and reproducibility, ultimately cultivating a sustainable, thriving research community.”

To improve the structure of the manuscript, we have changed the organization of our manuscript by grouping related sections across four different domains: Teaching computational skills to produce reproducible research; Development and distribution of data and software; Implementation of reproducible research; and Incentivizing reproducible research. We also improved the schematics of Figure 1 to reflect the new section structure.

While the compact format for commentary papers limits our ability to provide many details on all topics,

we added some details to improve the clarity of certain points. For example, we now emphasize CODE CHECK as a promising tool; provide better examples for archival stability; and provide a better context for how the use of package managers can help reproducibility.

Reviewer Comment #2: The authors discuss new platforms for reproducible analysis, and specifically mention the CODECHECK project. However, this does not appear to actually be available for public usage; the linked web site reads more like a plan for future work than a description of an ongoing project. There are other, much better known platforms for this, such as Binder (which codecheck uses) and Code Ocean, so it was surprising that the authors would focus on a project that doesn't actually exist.

Authors' Response: In order to avoid confusion from readers, we changed the text as follows.

"We highlight CODE CHECK (https://sje30.github.io/codecheck/) as a promising platform to follow. CODE CHECK allows researchers to input code and data, and check that an analysis is reproducible. For verified analyses, CODE CHECK issues a time-stamped "certificate of reproducible computation", which a reviewer of a paper can consider without running the analyses themselves."

Reviewer Comment #3: I'm not sure that my friends in the library sciences would agree that Github is an "archivally stable" platform.

Authors' Response: We thank the reviewer for raising this issue. We have changed the text in the paper as follows.

"Code used in a published analysis should be hosted on an archivally stable platform such as Software Heritage Archive (https://archive.softwareheritage.org/) or Zenodo (https://zenodo.org/) (Figure 1c)."

Reviewer Comment #4: I worry about promoting a small number of specific platforms, such as Stencilia - in part because it's not clear how long-lived these platforms will be, and also because this is a very fast moving area, so any recommendations are likely to be outdated within a short period.

Authors' Response: We listed specific examples to provide concrete examples to the readers. We agree these solutions may be short-lived. Therefore we have changed the discussion section as follows.

"The platforms listed in this paper are provided for illustration. Given this is a fast-moving area, some of our recommendations are likely to be outdated within a short period and other short-lived. We acknowledge that new platforms may appear soon. Therefore we have created a dynamic version11 of this paper to reflect new platforms, as an example to command scientific rigor and reproducibility ourselves. The dynamic version was compiled in markdown and includes an extended list of references."

Reviewer Comment #5: I am not sure why conda is listed under "reproducible workflows" as it is really more of a software distribution and package management system - it doesn't specifically do anything regarding workflows. Similarly for bioconda. There are also many different workflow systems, and focusing only on Galaxy and CWL seems too limiting.

Authors' Response: We have removed Bioconda and Conda from the list of reproducible workflows. We

have also changed the text in the paper as follows.

“Reproducibility can be enhanced with workflow-specific platforms, such as Galaxy and Tensorflow (for machine learning), and workflow standards, such as CWL (Common Workflow Language) (Table 1).”

We also changed the text to list conda and bioconda as package managers, and added the following sentence.

“Hosting software tools on package managers allows users to easily install software with more straightforward commands and automatically acquire resolutions for software dependencies. Examples of package managers are Conda and Bioconda (Table 1).”

Close
