# Supplementary material for: Recommendations to enhance rigor and reproducibility in biomedical research
Source: Gigascience. 2020 Jun 1;9(6):giaa056. doi: 10.1093/gigascience/giaa056 (PMC7263079; doi:10.1093/gigascience/giaa056)
Supplement: giaa056_Response_to_Reviewer_Comments_Revision_1 [file giaa056_response_to_reviewer_comments_revision_1.pdf]

Author's Response To Reviewer Comments

Close

May 05, 2020

Dear Dr. Scott Edmunds,

Thank you very much for considering our paper, entitled "Recommendations to enhance rigor and reproducibility in biomedical research," for publication at GigaScience. We are grateful to the reviewers for their careful assessment of the manuscript and the detailed and constructive comments that they provided. We believe that addressing these comments produced a much stronger manuscript that will hold more value for the scientific community.

Following the editor's comments, we have made changes listed below as a point-by-point response.

Sincerely,

Serghei Mangul, Ph.D.
Assistant Professor
Titus Department of Clinical Pharmacy
USC School of Pharmacy
University of Southern California

Response to Editor's Comments

Editor's Comment #1: Can you list ORCID IDs for any authors that have them.

Authors' Response: We have listed the authors' ORCID in the manuscript.

Editor's Comment #2: I agree with the reviewer that this is probably a bit early to promote, and it doesn't really fit in this section either. The first paper with a certificate has just been published, and other than the website there isn't really anything concrete to cite yet as Stephen Eglen hasn't written this up yet. Easier to delete.

Authors' Response: We have deleted the paragraph about CODE CHECK.

Editor's Comment #3: If you plug RRIDs you probably should add RRIDs to any registered software you discuss. e.g. Galaxy , RRID:SCR_006281, Binder (RRID:SCR_016437), CWL (RRID:SCR_015528). At least in this table.

Authors' Response: We have listed the RRIDs for the registered software in Table 1.

Editor's Comment #4: There are a huge number of workflow systems you could highlight here, but I don't know if you want to add Snakemake to the list, as that seems quite de rigeur at the moment, and the python community like it.

Authors' Response: We have added Snakemake to the list of Reproducible Workflows tools in Table 1 as follows.

"Snakemake (https://snakemake.readthedocs.io/en/stable/, RRID:SCR_003475) is a tool to create reproducible and scalable data-analyses workflows, with a language based on python. Snakemake makes it easier to execute data analyses on different environments without modification on the workflow definition."

Editor's Comment #5: F1000 had more basic R/ggplot2 examples since 2014 (https://blog.f1000.com/2014/09/09/living-figures-interview/), and has had examples with Plotly since 2017. This is more sophisticated, but if you want to keep this example you should tweak the wording so it sounds less like a first. Technically it was also a demonstration of a reproducible view of an existing eLife article. I would suggest something like: In 2018, eLife published a demonstration of a dynamic and code-based reproducible peer-reviewed paper, using the Stencila platform and Binder.

Authors' Response: We have changed the text according to the Editor's suggestion.

"In 2018, eLife published a demonstration of a dynamic and code-based reproducible peer-reviewed paper, using the Stencila platform and Binder (Table 1). This approach enables data and analysis to be fully reproducible by the reader and challenges the traditional static representation of results using PDF or HTML formats."

Editor's Comment #6: Need to remove this final reference and just include the URL. Can include this in the Acknowledgements section or an Editor's Note if that fits better. And as the paper is forkable/contributable to by others do you want to encourage others to contribute?.

Authors' Response: We have moved the referred statements to the Acknowledgements section. We have also removed the reference and added the URL at the end of the Conclusion section as follows.

"Given this is a fast-moving area, some of our recommendations are likely to be outdated within a short period and other short-lived. We acknowledge that new platforms may appear soon (https://github.com/Mangul-Lab-USC/enhancing_reproducibility)."

"Acknowledgments
We thank Dr. Lana Martin for helping with the design of Figure 1 and constructive discussions and comments on the manuscript. A dynamic version of this paper reflects new platforms https://github.com/Mangul-Lab-USC/enhancing_reproducibility, as an example to command scientific rigor and reproducibility ourselves. This dynamic version was compiled in markdown and includes an extended list of references. We encourage others to contribute to our repository."

Editor's Comment #7: Need the article number/DOI, so ideally should cite:
Piccolo SR, Frampton MB. Tools and techniques for computational reproducibility. Gigascience. 2016;5(1):30. doi:10.1186/s13742-016-0135-4

Authors' Response: We have edited the reference as follows.
"Piccolo SR, Frampton MB. Tools and techniques for computational reproducibility. GigaScience. 2016;5(1):30. doi:10.1186/s13742-016-0135-4"

Close
