# Supplementary material for: Recommendations to enhance rigor and reproducibility in biomedical research
Source: Gigascience. 2020 Jun 1;9(6):giaa056. doi: 10.1093/gigascience/giaa056 (PMC7263079; doi:10.1093/gigascience/giaa056)
Supplement: giaa056_Reviewer_1_Report_Original_Submission — Russell Poldrack -- 3/9/2020 Reviewed [file giaa056_reviewer_1_report_original_submission.pdf]

Reviewer Report

Title: Recommendations to enhance rigor and reproducibility in biomedical research

Version: Original Submission **Date:** 3/9/2020

Reviewer name: Russell Poldrack

Reviewer Comments to Author:

This paper aims to lay out a set of recommendations for enhancing the rigor and reproducibility of research across biomedicine. There is not much to specifically disagree with in the paper, and there are some points that I really like, such as the point that closed source/commercial software impairs reproducibility, the need for reproducible workflows, and the need for software engineers as part of research teams. At the same time, I don't feel that the paper really adds much to what is already out there - for example, in the many "ten simple rules" papers in PLOS Computational Biology. I also don't feel like the paper is organized very well; it seems to jump around quite a bit, and never gets into enough detail to really nail any of its points. I would suggest that the authors narrow their focus and build a clearer explication of their points in more detail.

There are also a number of specific points that I found problematic

1. The authors discuss new platforms for reproducible analysis, and specifically mention the CODECHECK project. However, this does not appear to actually be available for public usage; the linked web site reads more like a plan for future work than a description of an ongoing project. There are other, much better known platforms for this, such as Binder (which codecheck uses) and Code Ocean, so it was surprising that the authors would focus on a project that doesn't actually exist.
2. I'm not sure that my friends in the library sciences would agree that Github is an "archivally stable" platform.
3. I worry about promoting a small number of specific platforms, such as Stencilia - in part because it's not clear how long-lived these platforms will be, and also because this is a very fast moving area, so any recommendations are likely to be outdated within a short period.
4. I am not sure why conda is listed under "reproducible workflows" as it is really more of a software distribution and package management system - it doesn't specifically do anything regarding workflows. Similarly for bioconda. There are also many different workflow systems, and focusing only on Galaxy and CWL seems too limiting.

Level of Interest

Please indicate how interesting you found the manuscript: Choose an item.

Quality of Written English

Please indicate the quality of language in the manuscript: Choose an item.

Declaration of Competing Interests

Please complete a declaration of competing interests, considering the following questions:

- Have you in the past five years received reimbursements, fees, funding, or salary from an organisation that may in any way gain or lose financially from the publication of this manuscript, either now or in the future?
- Do you hold any stocks or shares in an organisation that may in any way gain or lose financially from the publication of this manuscript, either now or in the future?
- Do you hold or are you currently applying for any patents relating to the content of the manuscript?
- Have you received reimbursements, fees, funding, or salary from an organization that holds or has applied for patents relating to the content of the manuscript?
- Do you have any other financial competing interests?
- Do you have any non-financial competing interests in relation to this paper?

If you can answer no to all of the above, write 'I declare that I have no competing interests' below. If your reply is yes to any, please give details below.

I declare that I have no competing interests'

I agree to the open peer review policy of the journal. I understand that my name will be included on my report to the authors and, if the manuscript is accepted for publication, my named report including any attachments I upload will be posted on the website along with the authors' responses. I agree for my report to be made available under an Open Access Creative Commons CC-BY license (http://creativecommons.org/licenses/by/4.0/). I understand that any comments which I do not wish to be included in my named report can be included as confidential comments to the editors, which will not be published.

Choose an item.

To further support our reviewers, we have joined with Publons, where you can gain additional credit to further highlight your hard work (see: https://publons.com/journal/530/gigascience). On publication of this paper, your review will be automatically added to Publons, you can then choose whether or not to claim your Publons credit. I understand this statement.

Yes Choose an item.
